# Evolving changes in M-protein and hemoglobin as predictors for progression of smoldering multiple myeloma

**DOI:** 10.1038/s41408-018-0144-x

**Published:** 2018-11-08

**Authors:** Shebli Atrash, Myra Robinson, Daniel Slaughter, Amanda Aneralla, Taylor Brown, Jordan Robinson, Ami Ndiaye, Chelsea Sprouse, Qing Zhang, James T. Symanowski, Reed Friend, Peter M. Voorhees, Saad Z. Usmani, Manisha Bhutani

**Affiliations:** 1 Department of Hematologic Oncology and Blood Disorders, Levine Cancer Institute/Atrium Health, Charlotte, NC USA; 2 Department of Cancer Biostatistics, Levine Cancer Institute/Atrium Health, Charlotte, NC USA

Smoldering multiple myeloma (SMM) comprises a spectrum of distinct entities with varying risks of clonal evolution, leading to rapid progression to multiple myeloma in some patients and not in others. Fifty percent of patients with SMM develop multiple myeloma within the first 5 years, 25% behave like monoclonal gammopathy of undetermined significance with low risk of progression, and 25% of patients would never progress^1^. The standard practice of “wait and watch” leaves SMM patients with the potential risk of developing morbid complications from progression to multiple myeloma. On the other hand, early treatment for some of these patients may not impact overall survival but rather negatively impact quality of life. Therefore, it is essential to clearly identify “rapidly progressing” SMM patients and offer them early treatment on clinical trials.

Several natural history studies have developed models to risk stratify SMM patients into low, intermediate, and high risk of progression based on variables measured at the time of diagnosis. The most commonly used Mayo clinic^2^ model depends on monoclonal—(M) protein levels, free light chain (FLC) ratio, and bone marrow plasma cell percentage (BMPC), whereas the PETHEMA Spanish group^3^ model uses flow-cytometry to define the proportion of aberrant plasma cells in the marrow, and the presence of immunoparesis. Mayo Clinic proposed yet another risk model with revised cutoffs for markers measured at diagnosis^4^. These models do not consider chronological changes in biomarkers that may predict progression.

The risk of progression for patients with SMM is not uniform over time. Based on the pattern of evolution of the M-protein during the clinical course, we may identify two types of SMM: the evolving cases, characterized by a progressive increase in M-protein and the non-evolving cases with stable M-protein that abruptly increases when patients develop active multiple myeloma. Rosinol et al.^5^ showed evolving type of SMM patients characterized by a constant increase in M-protein at each visit had a shorter time to progression (TTP) than the non-evolving type with long-lasting stable serum or urinary M-protein (median of 1.3 vs. 3.9 years, respectively). Similarly, the Southwest Oncology Group study showed that six SMM patients in whom M-protein increased to ≥ 3 g/dL over a 3-month period had a progression risk of 33.3% at 2 years^6^. Another study from Spain assessed the predictive value of the evolving pattern of serum M-protein, defined as a progressive increase of at least 10% in the M-protein size within the first 12 months from diagnosis when baseline M-protein was ≥ 30 g/L or over a period of 3 years (with a progressive increase in the M-protein size in each of the annual measurements) in patients with an initial M-protein < 30 g/L^7^. Median time from recognition of evolving type to progression into symptomatic multiple myeloma was 1.1 years and progression rate at 3 years was 71%. Ravi et al. showed the added value of evolving change in biomarkers for classifying patients with more aggressive disease biology^8^. They described a new model by factoring in an evolving pattern in M-protein burden and hemoglobin to identify a subgroup of SMM patients at higher risk of early progression to symptomatic disease within the first 2 years of diagnosis. In this study, we examined the associations of change in hemoglobin and M-protein concentrations and risk of progression in our cohort of SMM patients.

Levine Cancer Institute’s (LCI) plasma cell disorder database was interrogated from January 2012 to May 2017 for all patients with SMM, defined by BMPC ≥ 10% and/or serum M-protein level ≥ 3 g/dL (or ≥ 500 mg/24 h in urine), and no CRAB features attributed to the plasma cell proliferative disorder. In an effort to be consistent with current IMWG 2014 diagnostic criteria for SMM^9^, we excluded those with BMPC ≥ 60%, or those who underwent an MRI examination at diagnosis and had > 1 focal lesion. We also excluded patients with less than 6 months of follow-up, or if they were treated for SMM within 6 months of their diagnosis. However, patients with involved to uninvolved serum FLC ratio ≥ 100 and involved FLC level > 10 mg/dL who did not meet other defining criteria of multiple myeloma were not excluded if they had been followed up without any signs of progression.

Clinical features and outcomes were captured. Evolving change in hemoglobin (eHb) was defined as ≥ 0.5 g/dl decrease within 12 months of diagnosis. Evolving change in serum M-protein level (eMP) was defined as ≥ 10% increase in M-protein and/or affected immunoglobulin (Ig) within the first 6 months of diagnosis (if M-protein ≥ 3g/dl) and/or a ≥ 25% increase in M-protein/Ig within the first 12 months, with a minimum required increase of 0.5 g/dl in M-protein and/or 500 mg/dl in Ig. TTP was derived for each patient as the time from diagnosis of SMM to diagnosis of multiple myeloma, based on CRAB features (proposed in 2014 IMWG consensus guidelines)^9^ or initiation of therapy for multiple myeloma. SMM patients without progression to multiple myeloma were censored at the date of the last assessment of their disease. Patients who died before documented progression to multiple myeloma were censored at their date of death. Time to event distributions were estimated using Kaplan Meier techniques and compared between groups using log rank tests. Hazard ratios were estimated using Cox proportional hazards regression. Additionally, a multivariable model for TTP was determined using model selection procedures, including backward elimination and forward selection with entry/elimination criteria of *p* = 0.10. Competing risk survival analysis methods were used to identify the risk of progression in the subjects with different numbers of risk factors, where death was treated as a competing risk event. Final results were compared between original^4^ and evolving-change^8^ Mayo clinic risk stratification models utilizing a generalized McNemar’s test of symmetry to determine if the two methods agreed. The data cutoff was August 3, 2018. The study was conducted in accordance with the Declaration of Helsinki and the Health Insurance Portability and Accountability Act guidelines of 1996.

**Fig. 1:**
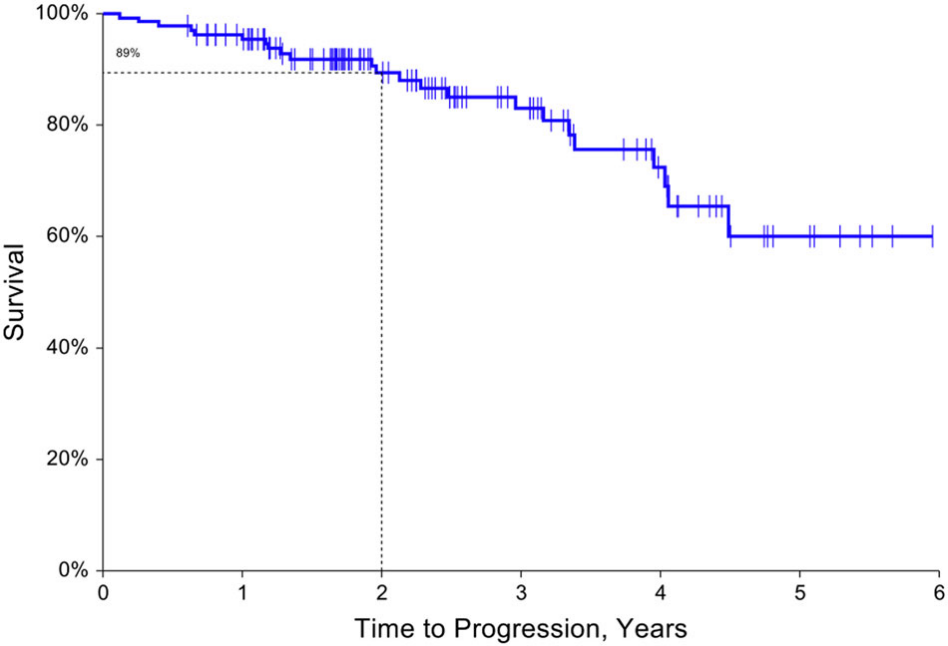
Cumulative incidence of progression for patients with smoldering multiple myeloma (*N* = 134). Cumulative incidence of progression was estimated with a death prior to progression considered as a competing risk event. There were 23 progression events. Median time to progression: not reached (LB of 95% CI: 4.1 years). Two-year progression-free rate: 89.4% (95% CI: 83.5–95.2%), and two-year progression rate: 10.6% (95% CI: 4.8–16.5%)

Of the 177 patients labeled as “SMM”, 134 patients met the eligibility criteria; 60 (44.8%) were male and 43 (32.1%) were African American (Table 1). Median follow-up was 28.2 months (range 7.3–71.4 months). Median age was 68 years (range 31–91 years), median age for Caucasian was 68 years and for African American was 66 years. Most patients had good-risk cytogenetics (56.7%), but 10.5% were high-risk cytogenetics. Of 133 patients with bone marrow biopsy results, 47 (35.3%) patients had 20–59% BMPC at diagnosis of SMM (Table 1). Seventeen patients (12.7%) met the criteria for eMP and 59 patients (44%) for eHb. No significant difference in distribution of number of risk factors was found between races. Median TTP for the whole group has not been reached. The estimated 2-year progression-free rate was 89.4% (95% CI: 83.5–95.2%, Fig. 1).

**Table 1:** Characteristics of patients with smoldering multiple myeloma (*n* = 134) and summary of important events

**Baseline characteristics at diagnosis**	***N***	**%**
*Gender*		
Male	60	44.8
Female	74	55.2
*Race*		
Caucasian	84	62.7
African American	43	32.1
Other	7	5.2
*Age, years*		
Median (range)	68	31–91
*Serum M-Protein, g/dL*		
<3	130	97.0
≥3	4	3.0
*Immunoglobulin subtype*		
IgG	93	69.4
IgA	23	17.2
IgM	1	0.8
LC	17	12.7
*Involved/uninvolved free light chain (FLC) ratio*		
<8	74	55.2
≥8	48	35.8
*Unknown*	12	9.0
*Hemoglobin, g/dL*		
Median (range)	12.4	7.1–16.2
Cytogenetic risk*		
Good	76	56.7
Intermediate	11	8.2
high	14	10.5
*Unknown*	33	24.6
*Beta-2-microglobulin mg/L*		
≤3.5	93	69.4
>3.5	16	11.9
*Unknown*	25	18.7
*LDH IU/L*		
Normal	85	63.4
High ( > upper limit of normal)	9	6.7
*Unknown*	40	29.9
*% Bone marrow plasma cells*		
10–19	86	64.2
20–59	47	35.1
*Unknown* ^**^	1	0.8
*eMP*		
Present	17	12.7
Absent	115	85.8
Not evaluable^***^	2	1.5
*eHb*		
Present	59	44.0
Absent	75	56.0
*Follow-up time, months*		
Median (range)	28.2	7.3–71.4
*Progression events to multiple myeloma (*N *=* *23)*		
Anemia	5	
Anemia/bone	6	
Anemia/hypercalcemia/bone	1	
Anemia, renal	2	
Bone	7	
Bone, hypercalcemia	1	
FLC ratio > 100	1	

*eHb* evolving pattern of hemoglobin, *eMP* evolving pattern of monoclonal protein

^*^Good risk: [normal cytogenetics, hyperdiploidy, t(11;14)]; Intermediate Risk: [t(4;14), t(6;14), del 13, others not in good or high risk]; high risk: [del 17p, t(14;16), t(14;20), Trisomy 1, amplification 1q21, complex cytogenetics, hypodiploidy

**One subject had unknown bone marrow plasma cell percentage at diagnosis, however the diagnosis was based on lambda light chain > 500 mg/24 h in urine

*** Patients were not evaluable if a second data point (within 12 months) could not be identified

Evolving patterns are defined as evolving m-protein/Ig or evolving Hb over the first 12 months of diagnosis as defined by Ravi et al.

**Fig. 2:**
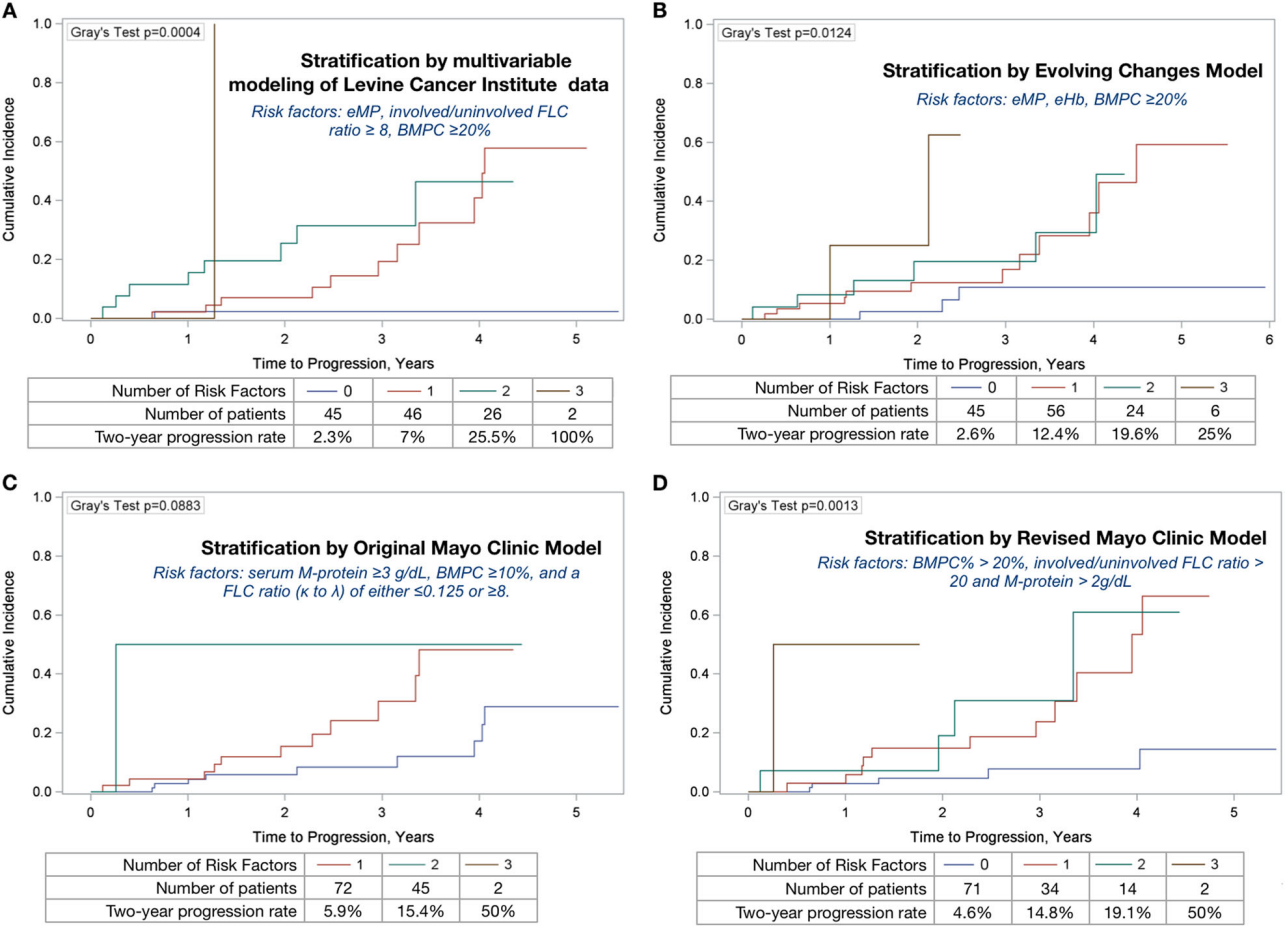
Cumulative incidence curves for time to progression, stratified by number of risk factors proposed in various risk models. **a**–**d** Cumulative incidence curves for time to progression, stratified by number of risk factors, estimated for each of the risk factor models: **a** Levine Cancer Institute model; **b** Evolving-Change model; **c** Traditional Mayo Clinic model; **d** Revised Mayo Clinic model. Gray’s test compared the cumulative incidence functions between risk factor groups within each model, with significance indicating a difference in the cumulative incidence distributions between the risk factor groups. Note, death was considered a competing risk event in the cumulative incidence estimation

*Risk factors based on multivariable modeling of LCI data:* We included following variables for TTP associations: age, sex, BMPC, M-protein level, FLC ratio, eMP, eHb, LDH, and beta-2-microglobulin (Table 2). The three risk factors: eMP, FLC ratio ≥ 8, and BMPC ≥ 20% were associated with TTP in univariable analysis (*p* = 0.006, *p* = 0.023, and *p* = 0.051, respectively), and remained significant in multivariable model (*p* = 0.003, *p* = 0.028, and *p* = 0.025, respectively) following model selection procedures (Table 2). With the three risk factors considered in LCI model, 45 patients (37.8%) were assigned with no risk factors, 46 (38.7%) had one risk factor, 26 (21.9%) had two risk factors, and 2 (1.7%) had three risk factors. Two-year progression rates were: 2.3, 7, 25.5, and 100% for no risk factor, one risk factor, two risk factors, and three risk factors, respectively (Fig. 2a).

**Table 2:** Univariable and multivariable cox proportional hazards models for risk factors in smoldering multiple myeloma

**Factor**	**Univariable results**		Multivariable results*	
	**HR**	***p*** **-value**	**HR**	***p*** **-value**
Age	1.02	0.420	-	-
Sex (male vs. female)	0.97	0.946	-	-
BMPC ( ≥ 20% vs. < 20%)	2.27	0.051	2.74	0.025
M-protein ( ≥ 3 vs. < 3 g/dL)	1.85	0.551	-	-
FLC ratio ( ≥ 8 vs. < 8)	2.79	0.023	2.66	0.028
eMP	3.90	0.006	4.47	0.003
eHb	2.04	0.097	-	-
LDH ( > upper limit of normal vs. normal)	2.51	0.233	-	-
Beta-2-microglobulin ( > 3.5 vs. ≤ 3.5 mg/L)	2.63	0.149	-	-

*BMPC* clonal bone marrow plasma cells, *95% CI* 95% confidence interval, *HR* hazard ratio, *FLC* free light chain, *eHb* evolving pattern of hemoglobin, *eMP* evolving pattern of monoclonal protein, *LDH* lactate dehydrogenase

*Note that the multivariable model is based on 88 subjects with fully available FLC ratio, eMP, and eHb data.

Evolving patterns are defined as evolving m-protein/Ig or evolving Hb over the first 12 mo of diagnosis as defined by Ravi et al.

*Applying Mayo Clinic evolving-change model*:^8^ Of 131 patients with data on all three variables (eMP, eHb, and BMPC ≥ 20%), 45 patients (34.4%) had zero risk factors, 56 (42.8%) had one risk factor, 24 (18.3%) had two risk factors, and 6 (4.6%) had three risk factors (Fig. 2b). There was a significant association between the number of risk factors and TTP (*p* = 0.007), with the risk of progression in patients with three risk factors 15.3 times the risk of progression in patients with zero risk factors. The estimated 2-year progression rate for patients with 0 risk factors was 2.6%, compared with 12.5% for patients with one risk factor, 20.6% for patients with two risk factors and 25% for three risk factors (Fig. 2b). In patients with eMP and eHb, the 2-year progression rate was 18.5% (95% CI: 2.4–46.5%), whereas for patients with both eMP and FLC ratio ≥ 8, the 2-year progression rate was 50% (95% CI: 2.3–88.1%).

*Applying Mayo Clinic’s traditional and revised cutoffs for risk factors at diagnosis*: We calculated the number of risk factors based on traditional Mayo clinic criteria^2^, which included serum M-protein ≥ 3 g/dL, BMPC ≥ 10%, and a FLC ratio (κ to λ) of either ≤ 0.125 or ≥ 8 (Fig. 2c). Of 117 patients with available data to calculate both the traditional model and the evolving-change model, 66 were in agreement, while 51 were discordant. The evolving-change model identified more low risk patients with “zero or one risk factors” compared with traditional Mayo Clinic model (90 vs. 55 patients). Applying the revised Mayo Clinic risk factors: BMPC% > 20%, involved FLC ratio > 20 and M-protein > 2 g/dL^4^, 121 patients were evaluable. Of these, 71 (58.7%) were assigned with no risk factors, 34 (28.1%) had one risk factor, and 16 had two or more risk factors (Fig. 2d). Two-year progression rates according to traditional and revised Mayo Clinic model are shown in Fig. 2c, d, respectively.

In this single institution, retrospective, cohort study, we validate the significance of evolving patterns of biomarkers for prognosticating SMM patients. In multivariable analysis, the presence of eMP, involved/uninvolved FLC ratio ≥ 8, and BMPC ≥ 20% emerged as significant risk factors for progression. Presence of none or one of these risk factors distinguished a population at a distinctly lower risk of progression than two or more risk factors. The 2-year progression rate for subjects with three risk factors was 25%, and for those with evolving changes in both eHb and eMP was 18.5%. These rates are comparatively much lower than described by Ravi et al.^8^ In their series, 21 patients with three risk factors had a 90.5% risk of progression, and individuals displaying eMP and eHb together had > 80% risk of progression to multiple myeloma within 2 years of diagnosis. The small sample size, few patients with high risk, and short follow-up could limit the generalizability of our results. Although this study involved more diversity with 43 African-American patients, we did not find significant difference between race and risk factor distribution. Our cohort inherently had very few high-risk SMM patients, as evaluated by traditional and revised Mayo clinic models utilizing different cutoffs for M-protein level, BMPC percentage, and FLC ratio.

Furthermore, eHb was not significantly associated with TTP (*p* = 0.097) in our study. Change in hemoglobin of ≥ 0.5 g/dl magnitude is prone to random fluctuation than are other markers. Several factors, such as measurement error, changes in hydration or nutritional status of patient, transient blood loss, or presence of end stage renal disease, can potentially affect hemoglobin variability with small fluctuations above or below the target range. Similarly, small changes in the measurements of M-protein level based on serum electrophoresis can pose challenges, although with standardization of laboratory techniques this marker can be readily integrated into prognostic models. Evolving changes in markers observed during observation period, combined with other static risk factors as FLC ratio and BMPC, allow for dynamic assessment of progression and should be validated in larger cohort.

The main limitation of our study is relatively small cohort of patients and overall few high-risk patients. Therefore, conclusions with respect to high-risk group cannot be drawn with confidence. Another limitation of our study is the lack of consistent longitudinal cytogenetics data to incorporate and evaluate for “spontaneous clonal evolution”, as described by Bolli et al.^10^ Nonetheless, our model incorporating eMP, FLC ratio ≥ 8, and BMPC ≥ 20% clearly identifies SMM patients with low risk of progression. Patients with none or one risk factor (low-risk group) are candidates for optimized follow-up, while patients with eHb and eMP should undergo restaging bone marrow biopsy and imaging to validate progression. More studies are required to incorporate evolving changes in dynamic markers, including evolution of genomic profiles to better prognosticate high-risk SMM patients that could be considered for clinical trials aiming to prevent progression.
